# Universal Behavior of the Coulomb-Coupled Fermionic Thermal Diode

**DOI:** 10.3390/e24121810

**Published:** 2022-12-12

**Authors:** Shuvadip Ghosh, Nikhil Gupt, Arnab Ghosh

**Affiliations:** Department of Chemistry, Indian Institute of Technology Kanpur, Kanpur 208016, India

**Keywords:** quantum thermodynamics, quantum dots, master equation, coulomb blockade, thermal diode

## Abstract

We propose a minimal model of a Coulomb-coupled fermionic quantum dot thermal diode that can act as an efficient thermal switch and exhibit complete rectification behavior, even in the presence of a small temperature gradient. Using two well-defined dimensionless system parameters, universal characteristics of the optimal heat current conditions are identified. It is shown to be independent of any system parameter and is obtained only at the mean transitions point “−0.5”, associated with the equilibrium distribution of the two fermionic reservoirs, tacitly referred to as “*universal magic mean*”.

## 1. Introduction

Heat management devices have attracted recent interest in nanoscale systems; they prevent overheating due to heat flow in desired areas of electronic circuits [1,2,3,4,5,6,7,8,9,10,11,12]. Following the theoretical proposal of a quantum dot thermal diode by Roukola and Ojanen [13], a number of studies have been carried out to achieve the rectification effect using simple quantum systems [14,15,16,17,18,19,20,21,22,23,24,25,26,27,28,29,30,30]. Yet, most of the works conducted to date rely on either temperature gradients of bosonic reservoirs or different coupling strengths between the system and the bath to break the inversion symmetry of the overall system [30]. For example, Werlang et al. [19] explored heat transport under the influence of strong coupling between two spins interacting with their respective bosonic baths. Miranda et al. [26] identified similar diode characteristics in the presence of different excitation frequencies between the coupled spins. Based on two- and four-terminal quantum dot setups, Tesser et al. [30] recently explored the roles of level degeneracy and temperature bias. However, much less attention is paid to achieving the rectification effect by means of the statistical properties of the reservoir. Here, we provide a general framework to capture the invariant aspect of the fermionic diodes in terms of two dimensionless physical parameters and statistical distribution of the reservoir—the *uniqueness* of which is independent of the system energy levels, interaction strength, bath spectrum, its temperature, and chemical potentials.

To showcase our findings, we considered a Coulomb-coupled quantum dot system and studied the interaction with two fermionic reservoirs with different temperatures and chemical potential. We find that, while the temperature gradient governs the overall heat flow direction, the magnitude of the heat current is primarily controlled by the chemical potential gradient. In contrast to the bosonic counterpart, the fermionic rectifier allows us to control the heat current and switching effects in a much more efficient way, even in the presence of tiny temperature differences. Most remarkably, we identified the universal nature of complete, partial, and no rectification conditions that are valid for all Coulomb-coupled fermionic diodes.

The present work is organized as follows: We introduce the model and dynamics in Section 2, and the steady-state heat current in Section 3. The microscopic picture behind the thermodynamically consistent heat flow direction is summarized in Section 4. Universal characteristics based on two dimensionless parameters are presented in Section 5, and the role of an efficient thermal switch and the ideal rectification effects are discussed in Section 6. Finally, we conclude in Section 7.

## 2. Model and Dynamics

**Figure 1:**
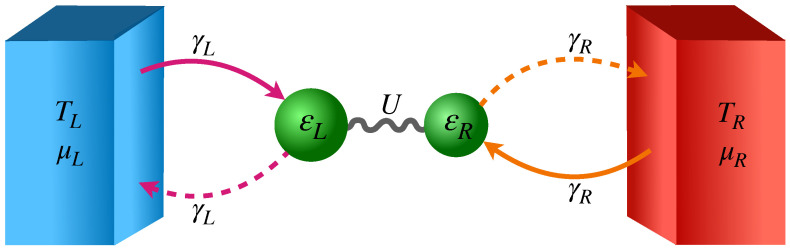
Coulomb-coupled QDs connected with fermionic reservoirs through sequential tunneling.

Our model consists of two quantum dots (QDs) that are strongly and capacitively coupled to each other and interact through a long-range Coulomb force so that they can exchange energy (but no particles). We consider this model following References [13,27,30,31,32], who recently introduced the study of thermal diodes and transistor effects in a wide variety of Coulomb blockade quantum dot devices. We further assume that each QD is tunnel-coupled to its fermionic reservoir [33]. While the electron transports between QDs are forbidden due to the Coulomb blockade [31], electron tunneling between QDs and their respective reservoirs permit heat flow from one reservoir to another through the coupled QD system. Within sequential tunneling [32] under the Coulomb blockade regime, each QD can only have two levels with the occupation number as either zero or one. The two QDs, as well as the temperature and chemical potential of the baths to which they are connected, are labeled by indices *L* and *R* [Figure 1]. The Hamiltonian of the coupled QDs is then given by,

(1) $$\begin{array}{c} H_{D}=\sum_{\alpha =L,R}\varepsilon_{\alpha }d_{\alpha }^{†}d_{\alpha }+\sum_{\alpha ,\beta =L,R;\alpha \neq \beta }U_{\alpha \beta }d_{\alpha }^{†}d_{\alpha }d_{\beta }^{†}d_{\beta }. \end{array}$$

Here, $\varepsilon_{\alpha }$ is the lowest single-particle energy of QDs. Without loss of any generality, we further assume $\varepsilon_{L}<\varepsilon_{R}$. Here, $U_{\alpha \beta }$ is the positive Coulomb interaction energy between the electrons in different QDs, and $d_{\alpha }^{†}\left(d_{\alpha }\right)$ denotes the creation (annihilation) operator for the α-th QD, whose eigenstates are |0〉 and |1〉 with eigenvalues 0 and $\varepsilon_{\alpha }$, respectively. Since the interaction energy in Equation (1) is diagonal in the eigenbases of the individual QDs, the eigenstates of $H_{D}$ w.l.o.g can be written in terms of the eigenstates of the two QDs, in decreasing energy order, as |1〉=|00〉,|2〉=|10〉,|3〉=|01〉,|4〉=|11〉. For details, please refer to References [13,31,32]. The Hamiltonian of the α-th fermionic reservoir is defined as $H_{R}^{\alpha }=\sum_{k}\left(\varepsilon_{k}-\mu_{\alpha }\right)c_{\alpha k}^{†}c_{\alpha k}$ [30], where $\varepsilon_{k}$ is the energy of the non-interacting reservoir electron, the continuous wavenumber *k*, $\mu_{\alpha }$ is the chemical potential, and $c^{†}\left(c\right)$ represents the creation (annihilation) operator of the electron reservoir. The coupling between QDs and the respective reservoir is described by the tunneling Hamiltonian, $H_{T}^{\alpha }=\sum_{k}\left(t_{\alpha k}c_{\alpha k}^{†}d_{\alpha }+t_{\alpha k}^{*}d_{\alpha }^{†}c_{\alpha k}\right)$, where $t_{\alpha k}$ is the tunneling amplitude. Interaction within sequential tunneling approximation imposes restrictions on simultaneous tunneling of more than one electron at a time [13,27,30,31]. Consequently, there are, in total, four authorized transitions: the left reservoir (*L*) induces transitions between 1↔2 and 3↔4, while the right bath (*R*) drives transitions between 1↔3 and 2↔4. We define transition energies $\omega_{ij}=\epsilon_{i}-\epsilon_{j}$, for i>j, where $\epsilon_{k}$ is the eigenvalue of $H_{D}$ for the eigenstate |k〉. In the present case, they read as $\omega_{21}=\varepsilon_{L}$, $\omega_{42}=\varepsilon_{R}+U$, $\omega_{43}=\varepsilon_{L}+U$, and $\omega_{31}=\varepsilon_{R}$. The rates at which the above transitions occur are computed using the Lindblad master equation [34,35].

We implement the strong-coupling formalism following References [19,36,37,38,39,40,41] to arrive at the master equation describing the time evolution of the density matrix ρ of the coupled QDs system (See Appendix A)

(2) $$\frac{d\rho }{dt}=-\frac{i}{\hbar }\left[H_{D},\rho \right]+L_{L}\left[\rho \right]+L_{R}\left[\rho \right],$$

under Born, Markov, and the secular approximation [19,21,34]. It is important to note here that the strong coupling formalism refers to the coupling between the dots, while the system–bath coupling is still assumed to be weak so that the Born–Markov approximation can safely be implemented [19,36,37,38,39,40,41]. This implies that the Lindbladians are obtained on the basis of the eigenstates of the full system Hamiltonian $H_{D}$. Thus, the dissipation mechanism of each QD depends not only on the coupling to its own bath but also on the coupling between QDs, which is necessary for accurately describing the heat flow and rectification effects over a wide range of system parameters as considered below.

## 3. Evaluation of Steady State Heat Current

**Figure 2:**
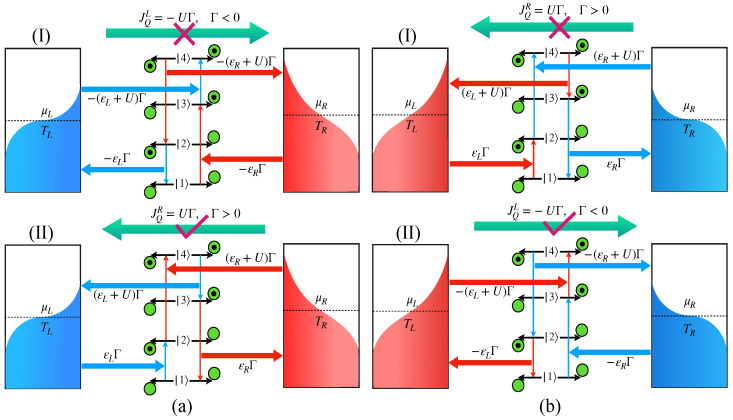
Thermodynamically consistent and inconsistent heat flow directions; probable paths for the transition cycle under (**a**) $T_{L}<T_{R}$; (**b**) $T_{R}<T_{L}$. In both conditions, Path-I is initiated by the hot bath and Path-II is initiated by the cold bath. Although Path-I seems to be the natural heat flow direction, the transition cycle actually encompasses Path-II in both limits, following the laws of thermodynamics.

**Figure 3:**
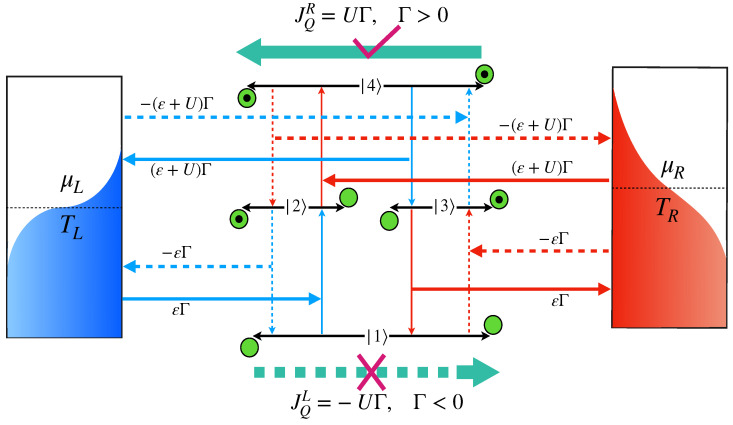
Thermodynamically consistent transition cycle for $T_{R}>T_{L}$ in case of $\varepsilon_{L}=\varepsilon_{R}=\varepsilon$; the first excitation from |1〉 must be guided by a cold bath (Path-II: solid lines), instead of a hot bath (Path-I: dotted lines).

In the present model, particles cannot be exchanged between QDs as they interact only through the long-range Coulomb force. As a result, there is no particle flow in between reservoirs through the QD system [13,27,30,31,32]. So, energy is exchanged only in the form of heat [See Figure 2 and Figure 3]. The expression of the heat current can then be obtained using the standard procedure [42] starting from the von Neumann entropy of the system, defined as $S\left[\rho \left(t\right)\right]=-k_{B}\mathrm{Tr}\left[\rho \left(t\right)\ln\rho \left(t\right)\right]$. Upon taking the time derivative of the von Neumann entropy, one obtains

(3) $$\frac{d}{dt}S\left[\rho \left(t\right)\right]=-\sum_{\alpha =L,R}k_{B}\mathrm{Tr}\left\{L_{\alpha }\left[\rho \left(t\right)\right]\ln\rho \left(t\right)\right\},$$

where we have used the master Equation (2) and $\mathrm{Tr}[\dot{\rho }]=0$. The Sphon inequality [43] in the form of the second law of thermodynamics [44] for any Lindblad superoperator L can be written as $\mathrm{Tr}\{L\left[\rho \left(t\right)\right]\left(\ln\rho \left(t\right)-\ln\rho_{ss}\right)\}⩽0$. Here, $\rho_{ss}$ is the steady-state population of the system, satisfying $L[\rho_{ss}]=0$. Denoting the stationary state of $L_{\alpha }$, as $\rho_{ss}^{\alpha }$, above, the inequality can be applied to both terms of the sum in Equation (3), yielding

(4) $$\sum_{\alpha =L,R}\mathrm{Tr}\left\{L_{\alpha }\left[\rho \left(t\right)\right]\left(\ln\rho \left(t\right)-\ln\rho_{ss}^{\alpha }\right)\right\}⩽0.$$

From Equations (3) and (4), we can write

(5) $$\begin{array}{c} \frac{d}{dt}S\left[\rho \left(t\right)\right]+\sum_{\alpha =L,R}k_{B}\mathrm{Tr}\left[L_{\alpha }\left[\rho \left(t\right)\right]\ln\rho_{ss}^{\alpha }\right]\geq 0 \end{array}.$$

The above equation can be compared with the dynamical version of the second law given by [42,44]

(6) $$\frac{d}{dt}S\left[\rho \left(t\right)\right]-\sum_{\alpha =L,R}\frac{J_{Q}^{\alpha }\left(t\right)}{T_{\alpha }}\geq 0.$$

This allows us to identify the heat current (energy flow rate) $J_{Q}^{\alpha }\left(t\right)$ associated with the α-th reservoir as

(7) $$J_{Q}^{\alpha }\left(t\right)=-\frac{1}{\beta_{\alpha }}\mathrm{Tr}\left\{L_{\alpha }\left[\rho \left(t\right)\right]\ln\rho_{ss}^{\alpha }\right\}.$$

For the steady state operation, the master Equation (2) drives the system towards a Gibbs-like stationary state, characterized by [41,42]

(8) $$\rho_{ss}^{\alpha }=Z^{-1}\exp\left[-\beta_{\alpha }H_{D}\right],$$

where $Z=\mathrm{Tr}\{\exp\left[-\beta_{\alpha }H_{D}\right]\}$. For the detailed derivation of the steady state’s heat current, we refer readers to the review articles by Kosloff and Gelbwaser et al. [42,44]. Inserting Equation (8) into Equation (7), we obtain the general expression for the heat current, which, under a steady state condition, simplifies to

(9) $$J_{Q}^{\alpha }=\mathrm{Tr}\left\{L_{\alpha }\left[\rho_{ss}\right]H_{D}\right\}=\sum_{\omega_{ij}}\omega_{ij}\Gamma_{ij}^{\alpha }.$$

Here, the net decaying rate $\Gamma_{ij}^{\alpha }$ from |i〉 to |j〉 (i>j) is denoted as

(10) $$\begin{array}{cc} \Gamma_{ij}^{\alpha }= & -\Gamma_{ji}^{\alpha }\\ \equiv & \gamma_{\alpha }\left[1-f_{\alpha }\left(\omega_{ij}\right)\right]\rho_{ii}-\gamma_{\alpha }f_{\alpha }\left(\omega_{ij}\right)\rho_{jj}={\left[\Gamma_{ij}^{\alpha }\right]}_{↓}-{\left[\Gamma_{ij}^{\alpha }\right]}_{↑}. \end{array}$$

The first term represents the emission and the second term corresponds to absorption; $\gamma_{\alpha }$ is the bare tunneling rate between the dots and respective reservoirs [Figure 1]; $f_{\alpha }\left(\omega_{ij}\right)$ is the Fermi distribution function (FDF)

(11) $$\begin{array}{c} f_{\alpha }\left(\omega_{ij}\right)\equiv f_{\alpha }\left(\omega_{ij},T_{\alpha },\mu_{\alpha }\right)=\left[1+\exp(\frac{\omega_{ij}-\mu_{\alpha }}{k_{B}T_{\alpha }}\right){]}^{-1} \end{array},$$

corresponding to the transition energy $\omega_{ij}=\epsilon_{i}-\epsilon_{j}$ between eigenstates |i〉 and |j〉 controlled by the α-th reservoir. The tunneling of electrons into or out of QDs is primarily governed by FDF. In our model, there are four allowed transitions and both lead guides to two transitions each: the *L* lead drives transitions between |1〉↔|2〉 and |4〉↔|3〉, while the *R* lead controls |1〉↔|3〉 and |4〉↔|2〉 transitions. For the sake of convenience of our analysis, the corresponding FDFs are expressed in terms of two dimensionless parameters, the effective tunneling barrier ($\chi_{\alpha }=\left(\varepsilon_{\alpha }-\mu_{\alpha }\right)/U$) and dimensionless thermal energy ($\xi_{\alpha }=k_{B}T_{\alpha }/U$), as follows

(12) $$\begin{array}{cc} f_{L}\left(\omega_{21}\right)={\left[1+\exp\left(\frac{\varepsilon_{L}-\mu_{L}}{k_{B}T_{L}}\right)\right]}^{-1}={\left[1+\exp\left(\frac{\chi_{L}}{\xi_{L}}\right)\right]}^{-1}= & f_{L}^{1},\\ f_{L}\left(\omega_{43}\right)={\left[1+\exp\left(\frac{\varepsilon_{L}+U-\mu_{L}}{k_{B}T_{L}}\right)\right]}^{-1}={\left[1+\exp\left(\frac{\chi_{L}+1}{\xi_{L}}\right)\right]}^{-1}= & f_{L}^{2},\\ f_{R}\left(\omega_{31}\right)={\left[1+\exp\left(\frac{\varepsilon_{R}-\mu_{R}}{k_{B}T_{R}}\right)\right]}^{-1}={\left[1+\exp\left(\frac{\chi_{R}}{\xi_{R}}\right)\right]}^{-1}= & f_{R}^{1},\\ f_{R}\left(\omega_{42}\right)={\left[1+\exp\left(\frac{\varepsilon_{R}+U-\mu_{R}}{k_{B}T_{R}}\right)\right]}^{-1}={\left[1+\exp\left(\frac{\chi_{R}+1}{\xi_{R}}\right)\right]}^{-1}= & f_{R}^{2}. \end{array}$$

Now, our task is to first evaluate the full expression of $\Gamma_{ij}^{\alpha }$ at the steady state, and then find out the expression of the heat current. Under the steady state condition, the master Equation (2) is characterized by ${\dot{\rho }}_{ss}=0$, which reduces to

(13) $$\begin{array}{cc} \; & {\dot{\rho }}_{11}=0=\Gamma_{31}^{R}-\Gamma_{12}^{L};\;\;{\dot{\rho }}_{22}=0=\Gamma_{12}^{L}-\Gamma_{24}^{R},\\ & {\dot{\rho }}_{33}=0=\Gamma_{43}^{L}-\Gamma_{31}^{R};\;\;{\dot{\rho }}_{44}=0=\Gamma_{24}^{R}-\Gamma_{43}^{L}.\;\; \end{array}$$

Thus, at the steady state, all net transition rates become equal to Γ, i.e., $\Gamma_{31}^{R}=\Gamma_{12}^{L}=\Gamma_{24}^{R}=\Gamma_{43}^{L}\equiv \Gamma$. The four sets of equations in Equation (13) are not independent since $\sum_{i}\rho_{ii}=1$, which uniquely solves all of the state occupation probabilities as well as the heat current in terms of a single quantity Γ: $\Gamma_{31}^{R}=\Gamma_{12}^{L}=\Gamma_{24}^{R}=\Gamma_{43}^{L}\equiv \Gamma$. From Equation (9), we can then evaluate the general expression of the heat current at the steady state as follows

(14) $$\begin{array}{cc} J_{Q}^{R}=\varepsilon_{R}\Gamma_{13}^{R}-\left(\varepsilon_{R}+U\right)\Gamma_{42}^{R}= & -\varepsilon_{R}\Gamma +\left(\varepsilon_{R}+U\right)\Gamma =U\Gamma ,\\ J_{Q}^{L}=\varepsilon_{L}\Gamma_{12}^{L}-\left(\varepsilon_{L}+U\right)\Gamma_{43}^{L}= & \;\varepsilon_{L}\Gamma -\left(\varepsilon_{L}+U\right)\Gamma =-U\Gamma . \end{array}$$

Thus, we finally arrive at the explicit analytical expression of the steady state heat current as

(15) $$\begin{array}{c} J_{Q}^{L}=-J_{Q}^{R}=-U\Gamma . \end{array}$$

To find out Γ, we rewrite Equation (13) in terms of $f_{L(R)}^{1}$ and $f_{L(R)}^{2}$

(16) $$\begin{array}{cc} {\dot{\rho }}_{11}=\gamma_{R}\left[1-f_{R}^{1}\right]\rho_{33}-\left[\gamma_{R}f_{R}^{1}+\gamma_{L}f_{L}^{1}\right]\rho_{11}+\gamma_{L}\left[1-f_{L}^{1}\right]\rho_{22}=0, & \\ {\dot{\rho }}_{22}=\gamma_{L}f_{L}^{1}\rho_{11}-\left[\gamma_{L}\left(1-f_{L}^{1}\right)+\gamma_{R}f_{R}^{2}\right]\rho_{22}+\gamma_{R}\left[1-f_{R}^{2}\right]\rho_{44}=0, & \\ {\dot{\rho }}_{33}=\gamma_{R}f_{R}^{1}\rho_{11}-\left[\gamma_{L}f_{L}^{2}+\gamma_{R}\left(1-f_{R}^{1}\right)\right]\rho_{33}+\gamma_{L}\left[1-f_{L}^{2}\right]\rho_{44}=0, & \\ {\dot{\rho }}_{44}=\gamma_{L}f_{L}^{2}\rho_{33}-\left[\gamma_{R}\left(1-f_{R}^{2}\right)+\gamma_{L}\left(1-f_{L}^{2}\right)\right]\rho_{44}+\gamma_{R}f_{R}^{2}\rho_{22}=0. \end{array}$$

defined through Equation (12) and find out the steady state populations subject to the condition

(17) $$\rho_{11}+\rho_{22}+\rho_{33}+\rho_{44}=1.$$

Using Equation (16) and Equation (17), we can construct

(18) $$M\left[\begin{array}{c} \rho_{11}\\ \rho_{22}\\ \rho_{33}\\ \rho_{44} \end{array}\right]=\left[\begin{array}{c} 0\\ 0\\ 0\\ 1 \end{array}\right],$$

where,

(19) $$M=\left[\begin{array}{cccc} -[\gamma_{R}f_{R}^{1}+\gamma_{L}f_{L}^{1}] & \gamma_{L}\left[1-f_{L}^{1}\right] & \gamma_{R}\left[1-f_{R}^{1}\right] & 0\\ \gamma_{L}f_{L}^{1} & \left[\gamma_{L}\left(1-f_{L}^{1}\right)+\gamma_{R}f_{R}^{2}\right] & 0 & \gamma_{R}\left[1-f_{R}^{2}\right]\\ \gamma_{R}f_{R}^{1} & 0 & \left[\gamma_{L}f_{L}^{2}+\gamma_{R}\left(1-f_{R}^{1}\right)\right] & \gamma_{L}\left[1-f_{L}^{2}\right]\\ 1 & 1 & 1 & 1 \end{array}\right].$$

Solving the above equation, the expressions for the steady state populations $\rho_{ii}$ are

$$\begin{array}{cc} \rho_{11}=-\frac{1}{\left|M\right|}[ & \gamma_{L}{\gamma_{R}}^{2}\left\{\left(1-f_{L}^{1}\right)\left(1-f_{R}^{1}\right)\left(1-f_{R}^{2}\right)+\left(1-f_{L}^{2}\right)\left(1-f_{R}^{1}\right)f_{R}^{2}\right\}\\ + & \gamma_{R}{\gamma_{L}}^{2}\left\{\left(1-f_{L}^{1}\right)\left(1-f_{R}^{2}\right)f_{L}^{2}+\left(1-f_{L}^{2}\right)\left(1-f_{R}^{1}\right)\left(1-f_{L}^{1}\right)\right\}],\\ \rho_{22}=-\frac{1}{\left|M\right|}[ & \gamma_{L}{\gamma_{R}}^{2}\left\{f_{L}^{1}\left(1-f_{R}^{1}\right)\left(1-f_{R}^{2}\right)+f_{L}^{2}\left(1-f_{R}^{2}\right)f_{R}^{1}\right\}\\ + & \gamma_{R}{\gamma_{L}}^{2}\left\{f_{L}^{1}\left(1-f_{R}^{2}\right)f_{L}^{2}+\left(1-f_{L}^{2}\right)\left(1-f_{R}^{1}\right)f_{L}^{1}\right\}],\\ \rho_{33}=-\frac{1}{\left|M\right|}[ & \gamma_{L}{\gamma_{R}}^{2}\left\{\left(1-f_{L}^{2}\right)f_{R}^{1}f_{R}^{2}+\left(1-f_{L}^{1}\right)\left(1-f_{R}^{2}\right)f_{R}^{1}\right\}\\ + & \gamma_{R}{\gamma_{L}}^{2}\left\{f_{R}^{2}\left(1-f_{L}^{2}\right)f_{L}^{1}+\left(1-f_{L}^{2}\right)f_{R}^{1}\left(1-f_{L}^{1}\right)\right\}],\\ \rho_{44}=-\frac{1}{\left|M\right|}[ & \gamma_{L}{\gamma_{R}}^{2}\left\{f_{L}^{1}f_{R}^{2}\left(1-f_{R}^{1}\right)+f_{L}^{2}f_{R}^{1}f_{R}^{2}\right\}\\ + & \gamma_{R}{\gamma_{L}}^{2}\left\{f_{L}^{1}f_{R}^{2}f_{L}^{2}+\left(1-f_{L}^{1}\right)f_{L}^{2}f_{R}^{1}\right\}], \end{array}$$

where |M| stands for the determinant of the matrix M. Using the above expressions, we can evaluate the final expression of Γ as follows

(20) $$\Gamma =\frac{\gamma_{L}+\gamma_{R}}{\gamma_{L}\gamma_{R}}\left[\frac{f_{R}^{1}f_{R}^{2}f_{L}^{2}-f_{R}^{1}f_{R}^{2}f_{L}^{1}+f_{L}^{1}f_{L}^{2}f_{R}^{1}-f_{L}^{1}f_{L}^{2}f_{R}^{2}+f_{L}^{1}f_{R}^{2}-f_{L}^{2}f_{R}^{1}}{f_{L}^{1}f_{R}^{1}+f_{L}^{2}f_{R}^{2}-f_{L}^{1}f_{R}^{2}-f_{L}^{2}f_{R}^{1}-1}\right].$$

Using Equation (20), the final expression of the steady state heat current in terms of dimensionless parameters $\chi_{\alpha }$ and $\xi_{\alpha }$ can be written as

(21) $$J_{Q}^{L}=-J_{Q}^{R}=-\frac{U(\gamma_{L}+\gamma_{R})}{\gamma_{L}\gamma_{R}}\left[\frac{\exp\left(\frac{1}{\xi_{L}}\right)-\exp\left(\frac{1}{\xi_{R}}\right)}{X}\right],$$

where,

(22) $$\begin{array}{cc} X= & [\exp(\frac{1}{\xi_{L}})\left[2+\exp(-\frac{\chi_{R}}{\xi_{R}})+\exp(\frac{\chi_{L}}{\xi_{L}})+\exp\left(\frac{\chi_{L}}{\xi_{L}}-\frac{\chi_{R}}{\xi_{R}}\right)\right]\\ + & \exp\left(\frac{1}{\xi_{R}}\right)\left[2+\exp\left(-\frac{\chi_{L}}{\xi_{L}}\right)+\exp\left(\frac{\chi_{R}}{\xi_{R}}\right)+\exp\left(\frac{\chi_{R}}{\xi_{R}}-\frac{\chi_{L}}{\xi_{L}}\right)\right]\\ + & \exp\left(\frac{1}{\xi_{L}}+\frac{1}{\xi_{R}}\right)\left[\exp\left(\frac{\chi_{R}}{\xi_{R}}+\exp\frac{\chi_{L}}{\xi_{L}}\right)+\exp\left(\frac{\chi_{L}}{\xi_{L}}+\frac{\chi_{R}}{\xi_{R}}\right)\right]\\ + & \left[\exp\left(-\frac{\chi_{R}}{\xi_{R}}\right)+\exp\left(-\frac{\chi_{L}}{\xi_{L}}\right)+\exp\left(-\frac{\chi_{L}}{\xi_{L}}-\frac{\chi_{R}}{\xi_{R}}\right)\right]]. \end{array}$$

This is the exact analytical expression of heat current derived under the Born–Markov master equation. Since *U*, $\gamma_{L}$, $\gamma_{R}$ and *X* are all positive, it is immediately clear from Equations (21) and (22) that if $\xi_{R}>\xi_{L}$ ($T_{R}>T_{L}$), heat will flow from right to left ($J_{R}>0$) in accordance with Equation (15).

## 4. Microscopic Description of Heat Flow

Classically, heat flows according to the laws of thermodynamics, i.e., from high to low temperatures. Quantum mechanically, the flow of the heat current must be governed by a microscopic description, without violating the ultimate laws of thermodynamics [44,45,46,47,48].

Since the QDs are strongly coupled with each other, they form a four-level system as depicted in Figure 2, where the inter-dot transition is restricted due to the long-range Coulomb force. So, the energy transfer is only caused by the heat baths via the coupled states of the overall system [13,27,30,31,32]. For $T_{R}>T_{L}$, the natural heat flow direction will be from *R* to *L* with $J_{Q}^{R}>0$. In this case, the first excitation from the ground state must be guided by the cold bath, instead of a hot bath, which may appear paradoxical at first sight but makes the net transition rate Γ>0 as required by Equation (15). If the cycle follows the opposite path 1→3→4→2→1 [Figure 2(aI)], then $J_{Q}^{L}>0$, as the UΓ amount of energy should be delivered by the left bath to the right bath, which is in complete disagreement with the laws of thermodynamics. So, in order to be consistent with the laws of thermodynamics, the transition cycle must run in 1→2→4→3→1, initiated by the cold bath. This is seemingly paradoxical in the sense that, classically, we expect that, during the heat flow, energy is supplied by the hot bath and dumped into the cold bath. For the specific example, it is more favorable for the cold bath to make the 1→2 transition in Figure 2(aII), which costs $\varepsilon_{L}$ amount of energy than the 3→4 transition in Figure 2(aI), which requires $\left(\varepsilon_{L}+U\right)$ amount of energy. It is interesting to note that, for $T_{L}>T_{R}$, (i.e., $J_{Q}^{L}>0$, the heat flows from the left to the right following Figure 2(bII); the first transition is still mediated by the cold bath between 1→3, as it requires less energy ($\varepsilon_{R}$) than the 2→4 transition ($\varepsilon_{R}+U$) in Figure 2(bI).

It is important to emphasize that although we have taken $\varepsilon_{L}<\varepsilon_{R}$ to draw the schematic energy level diagram of Figure 2, the magnitude of the heat current, in particular, depends only on the two dimensionless parameters $\left\{\chi_{\alpha },\xi_{\alpha }\right\}$ via Equation (21). In the next section, we will explore the universal characteristics of the heat current solely based on these two dimensionless system parameters. For instance, Figure 3 shows that the basic principle behind the thermodynamically consistent transition cycle remains the same even in the case of $\varepsilon_{L}=\varepsilon_{R}$.

## 5. Universal Characteristic Due to Magic Means

**Figure 4:**
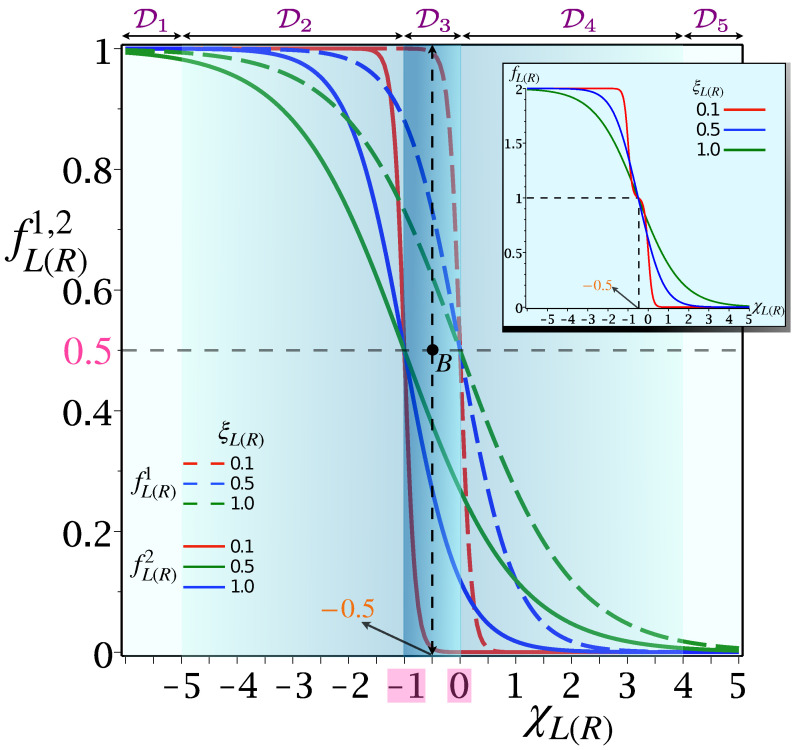
$D_{3}$ always spreads between transition points of $f_{L(R)}^{1}$ and $f_{L(R)}^{2}$; span of $D_{1[5]}$ and $D_{2[4]}$ are not fixed and strongly depend on the value of $\xi_{L(R)}$. The color gradients signify the magnitude of $|J_{Q}|$.

While the temperature gradient dictates the overall heat flow direction, chemical potential plays a very important role in determining the heat current’s magnitudes. To illustrate, we note that FDFs $f_{L(R)}^{1}$ and $f_{L(R)}^{2}$ in Equation (12), expressed in terms of dimensionless thermal energy $\xi_{L(R)}=k_{B}T_{L(R)}/U$ and the effective tunneling barrier $\chi_{L(R)}=\left(\varepsilon_{L(R)}-\mu_{\alpha }\right)/U$, are constrained by $0\leq f_{L(R)}^{2}<f_{L(R)}^{1}\leq 1$ and become 0.5 at $\chi_{L(R)}=0$ and −1, which correspond to $\mu_{L(R)}=\omega_{21(31)}=\varepsilon_{L(R)}$ and $\mu_{L(R)}=\omega_{43(42)}=\varepsilon_{L(R)}+U$, respectively. According to Equation (10), if $0\leq \{f_{L(R)}^{1},f_{L(R)}^{2}\}\leq 0.5$, it favors de-excitation and, equivalently, excitation is favored when $0.5\leq \{f_{L(R)}^{1},f_{L(R)}^{2}\}\leq 1$, for the corresponding transition. This is unique to fermionic reservoirs and allows us to implement a systematic analytical scheme solely based on the FDFs, and is in sharp contrast to its bosonic counterpart. The span of $f_{L(R)}^{1}$ and $f_{L(R)}^{2}$ as function $\chi_{L(R)}$, can thus be classified into five domains $D_{\left[1-5\right]}$ [Figure 4: Main] and the total FDF, defined as $f_{L(R)}=f_{L(R)}^{1}+f_{L(R)}^{2}$, is found to be spread between $0\leq f_{L(R)}\leq 2$ [Figure 4: Inset].

In domain $D_{1[5]}$, both $f_{L(R)}^{1}$ and $f_{L(R)}^{2}∼1\left[0\right]$, thus Equation (10) reduces to $\Gamma_{21/43}^{L}≃{\left\{\Gamma_{21/43}^{L}\right\}}_{↑[↓]}$ and $\Gamma_{31/42}^{R}≃{\left\{\Gamma_{31/42}^{R}\right\}}_{↑[↓]}$, i.e., only absorption (emission) is allowed in all four transitions; hence, the transition cycle can’t be completed [Figure 2], which results in vanishing $|J_{Q}|\to 0$. Now, $D_{2[4]}$ is characterized by $0.5\left[0\right]≲\left\{f_{L(R)}^{1},f_{L(R)}^{2}\right\}≲1\left[0.5\right]$; hence, excitation and de-excitation are favored for all four transitions yielding non-zero heat currents. On the contrary, domain $D_{3}$, parameterized by $-1\leq \chi_{L(R)}\leq 0$, is sharply defined between the transition points $f_{L(R)}^{2}$ and $f_{L(R)}^{1}$. As opposed to $D_{3}$, both $f_{L(R)}^{1}$ and $f_{L(R)}^{2}$ are closer to 1[0] in $D_{2[4]}$, so that the heat flux decreases in $D_{2[4]}$ relative to $D_{3}$. In $D_{3}$, both $f_{L(R)}^{1}$ and $f_{L(R)}^{2}$ take values, such that excitation from |1〉→|2〉(|2〉→|4〉) and de-excitation from |4〉→|3〉(|3〉→|1〉) can occur simultaneously at optimal rates. The condition becomes ideal at the midpoint of $D_{3}$ (point *B* in Figure 4), which corresponds to $\chi_{L(R)}=-0.5$. To find out, analytically, the optimal value of $\chi_{L(R)}$ for which $|J_{Q}|$ becomes maximum, first we have to differentiate $J_{Q}\equiv J_{Q}^{R}$ (Cf. Equation (21)) w.r.t. $\chi_{L(R)}$ and set that equal to zero. Now, by differentiating Equation (21) w.r.t $\chi_{R}$, for fixed $\left\{\chi_{L},\xi_{L},\xi_{R}\right\}$, we obtain,

(23) $$\begin{array}{cc} \; & {\left(\frac{\partial J_{Q}^{R}}{\partial \chi_{R}}\right)}_{\chi_{L},\xi_{L},\xi_{R}}\\ \; & =\frac{U(\gamma_{L}+\gamma_{R})}{\gamma_{L}\gamma_{R}}\left[\frac{\partial }{\partial \chi_{R}}\left\{\frac{f_{R}^{1}f_{R}^{2}f_{L}^{2}-f_{R}^{1}f_{R}^{2}f_{L}^{1}+f_{L}^{1}f_{L}^{2}f_{R}^{1}-f_{L}^{1}f_{L}^{2}f_{R}^{2}+f_{L}^{1}f_{R}^{2}-f_{L}^{2}f_{R}^{1}}{f_{L}^{1}f_{R}^{1}+f_{L}^{2}f_{R}^{2}-f_{L}^{1}f_{R}^{2}-f_{L}^{2}f_{R}^{1}-1}\right\}\right]\\ \; & =0. \end{array}$$

As, U>0 and $\gamma_{L},\gamma_{R}\neq 0$, Equation (23) implies that the only a non-trivial solution for maximizing $|J_{Q}|$ is equivalent to maximizing Γ, which is given by the criteria

(24) $$f_{R}^{1}+f_{R}^{2}=1.$$

It is clear from Equation (23) that the heat current vanishes under two limiting conditions: $\left(i\right)\;f_{R}^{1}=f_{R}^{2}=0;\;\left(ii\right)\;f_{R}^{1}=f_{R}^{2}=1$. Again, from Equation (12), we can write

(25) $$1\geq f_{\alpha }^{1}\geq f_{\alpha }^{2}\geq 0,\;\alpha =L,R.$$

So, Equation (25) signifies that, if $f_{\alpha }^{1}=0$ then $f_{\alpha }^{2}$ is certainly 0. Similarly, if $f_{\alpha }^{2}=1$ then $f_{\alpha }^{1}$ is certainly 1. In both cases, the heat current vanishes. Thus, the only condition for the nonzero heat current reduces to $f_{R}^{1}\neq 0$ and $f_{R}^{2}\neq 1$. Under these conditions, after solving Equation (24), we obtain the value of $\chi_{R}=-0.5$, for which $|J_{Q}|$ attains the maximum:

(26) $$\begin{array}{cc} \; & f_{R}^{1}=1-f_{R}^{2}\\ \mathrm{or},\; & \frac{1}{f_{R}^{1}}=\frac{1}{1-f_{R}^{2}}\\ \mathrm{or},\; & 1+\exp\left(\frac{\chi_{R}}{\xi_{R}}\right)=1+\exp\left(-\frac{\chi_{R}+1}{\xi_{R}}\right)\\ \mathrm{or},\; & \exp\left(\frac{\chi_{R}}{\xi_{R}}\right)=\exp\left(-\frac{\chi_{R}+1}{\xi_{R}}\right)\\ \mathrm{or},\; & \frac{\chi_{R}}{\xi_{R}}=-\frac{\chi_{R}+1}{\xi_{R}}\\ \mathrm{or},\; & \chi_{R}=-\chi_{R}-1\\ \mathrm{or},\; & \chi_{R}=-0.5. \end{array}$$

**Figure 5:**
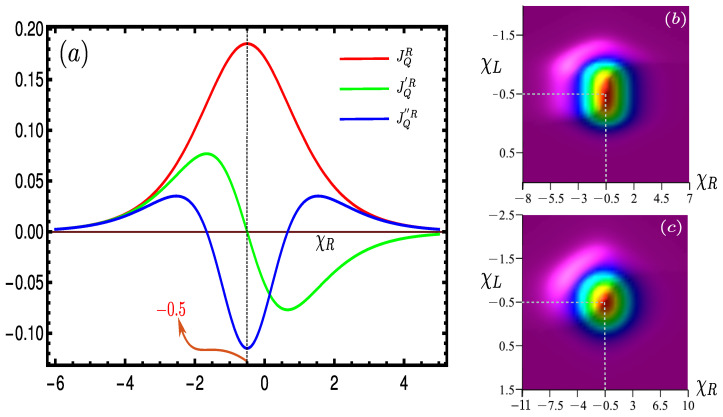
(**a**) Variation of heat current $J_{Q}^{R}$ (red line) with $\chi_{R}$ for fixed $\chi_{L}$, $\xi_{R}$ and $\xi_{L}$. The heat current becomes maximum at $\chi_{R}=-0.5$, which is supported by the plots of the first and second derivatives of the heat current; $J_{Q}^{\prime R}$ (green line) is zero and $J_{Q}^{\prime \prime R}$ (blue line) is negative at the magic mean point $\chi_{R}=-0.5$. The absolute value of the heat current $|J_{Q}|$ is plotted as a function of (**b**) $\left\{\chi_{L},\chi_{R}\right\}$ for $\xi_{R}=0.5$ and $\xi_{L}=0.2$; (**c**) $\left\{\chi_{L},\chi_{R}\right\}$ for $\xi_{R}=1$ and $\xi_{L}=0.5$.

So, the heat current will be maximum when $\chi_{R}=-0.5$ [Figure 5a]; similarly, when $\chi_{L}$ varies, $\chi_{L}=-0.5$ is the criteria for having the highest heat current. Hence, we can conclude that $|J_{Q}|$ is maximum when both $\chi_{L(R)}=-0.5$, irrespective of $\xi_{L(R)}$, which is supported by numerical simulations [Figure 5b,c] and also follows from analytically derived conditions $f_{L(R)}=1$ [Cf. Equation (24)] for the maximum heat current [Figure 4: Inset]. Now, putting these conditions in Equation (21), we can evaluate the exact analytical expression of the maximum heat current as

(27) $$\begin{array}{cc} \; & |J_{Q}{|}_{max}\\ \; & =\frac{U(\gamma_{L}+\gamma_{R})}{\gamma_{L}\gamma_{R}}|\frac{\sinh\left(\frac{1}{2\xi_{L}}-\frac{1}{2\xi_{R}}\right)}{2\left[1+\cosh\left(\frac{1}{2\xi_{R}}\right)+\cosh\left(\frac{1}{2\xi_{L}}\right)\right]+\cosh\left(\frac{1}{2\xi_{L}}-\frac{1}{2\xi_{R}}\right)+\cosh\left(\frac{1}{2\xi_{R}}-\frac{1}{2\xi_{L}}\right)}|\\ \; & \equiv {U|\Gamma |}_{max}. \end{array}$$

Several remarks are now in order:
  - The maximum heat current is obtained at $\chi_{L(R)}=-0.5$ and the magnitude only depends on the temperature of two leads, their tunneling rates, and the Coulomb interaction between the dots. Since −0.5 is the mean of 0 and −1, which are transition points of $f_{L(R)}^{1}$ and $f_{L(R)}^{2}$, respectively (point *B* in Figure 4: main), and this mean is also independent of other controlling parameters, we term this number “−0.5” as the “*universal magic mean*”. The significance of the point *B* lies in the fact that at the magic mean $\chi_{L(R)}=-0.5$, the chemical potential of the left (right) lead becomes exactly equal to the means of the transition energies $\omega_{21(31)}$ and $\omega_{43(42)}$ driven by that bath:
    (28) $$\begin{array}{cc} \; & \chi_{L(R)}=-0.5\\ \Rightarrow \; & \varepsilon_{L(R)}-\mu_{L(R)}=-0.5U\\ \Rightarrow \; & \mu_{L(R)}=\frac{2\varepsilon_{L(R)}+U}{2}\\ \Rightarrow \; & \mu_{L}=\frac{\omega_{43}+\omega_{21}}{2};\;\mu_{R}=\frac{\omega_{42}+\omega_{31}}{2}. \end{array}$$
    and, therefore, provides maximum control over the L(R) bath to guide both the absorption and decay simultaneously at the maximum Γ, with the resulting maximum $|J_{Q}|$.
  - With the increase of $\xi_{L(R)}$, $|J_{Q}|$ also spreads out, keeping the maxima point fixed at $\chi_{L(R)}=-0.5$. This precisely indicates that the maximum heat current will invariably be obtained at $\chi_{L(R)}=-0.5$, irrespective of all $\xi_{L,R}$ [Figure 5b,c]. The 3D plots along $\chi_{L}$ are squeezed for smaller values of $\xi_{L(R)}$ [Figure 5b] (assuming $\xi_{L}<\xi_{R}$, as in Figure 2), and if we increase $\xi_{L}\left(\xi_{R}\right)$, it expands along both positive and negative $\chi_{L(R)}$, leaving out the point of maxima intact at $\chi_{L(R)}=-0.5$ [Figure 5c]. This fundamental feature of the “magic mean” makes it truly *universal*.

## 6. Efficient Heat Current Modulator

**Figure 6:**
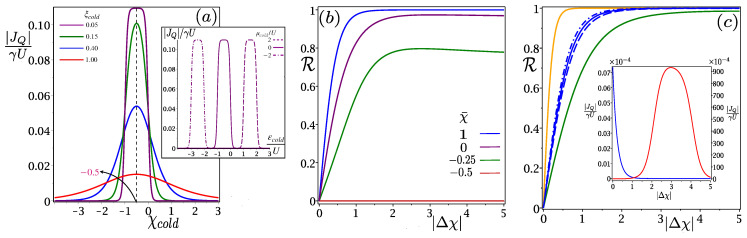
(**a**) Main: the thermal switching effect is obtained with the variation of the scaled heat current $|J_{Q}|/\gamma U$ w.r.t $\chi_{cold}$ and becomes more prominent with $\xi_{cold}\ll 1$ as the domain’s ON and OFF become more precise. Inset: expressing the switching effect in terms of the controllable parameter $\varepsilon_{L(R)}$. (**b**) No rectification (R=0) is obtained irrespective of the value of Δξ, if $\bar{\chi }=-0.5$ or Δχ=0 (red line). Partial (purple and green lines) and complete (blue line) rectifications are obtained for nonzero |Δχ| and $\bar{\chi }$ deviating from the magic mean −0.5. (**c**) Main: $\xi_{cold}$ (orange and green lines) have a stronger dependence on rectification than the $\xi_{hot}$ (blue dashed and dashed-dot lines) for an equal change in |Δξ|, where the solid blue line corresponds to the blue curve of (**b**). Inset: the magnitude of the heat current decreases by an amount $10^{-6}$ upon reversing the temperature gradient for the blue curve in (**b**).

With the increase of $\xi_{L(R)}$, $D_{1[5]}$ becomes narrower while $D_{2[4]}$ spreads out, without affecting $D_{3}$ [Figure 4]. As a consequence, $|J_{Q}|$ spreads out with $\xi_{L(R)}$, keeping the point of maxima fixed at $\chi_{L(R)}=-0.5$. Now, if $\xi_{L(R)}\ll 1$, then the span of $D_{2[4]}$ is very small compared to $D_{1[5]}$, while domain $D_{3}$ always remains in between $-1\leq \chi_{L(R)}\leq 0$. So, there are effectively two domains: (I) Domain ON, where $|J_{Q}|\neq 0$ and (II) Domain OFF, where $|J_{Q}|=0$ and the switching effect becomes more prominent if $\xi_{L(R)}\ll 1$. Thus, we can operate our model as an efficient thermal switch to the on–off heat current just by shifting the domain from ON to OFF through the change of $\chi_{L(R)}$ or, in turn, the controllable experimental parameter $\varepsilon_{L(R)}$ [Figure 6a]. This is the basic underlying principle behind the switching effect for each Coulomb-coupled fermionic thermal diode. It becomes more prominent for a smaller cold bath temperature $\xi =k_{B}T/U$ and can be achieved more easily by varying *U*, instead of lowering *T* to a smaller value.

Finally, the model operates as an efficient thermal diode with the rectification factor (R) approaching 1, even in the presence of a low-temperature gradient. This produces a clear advantage over all previously proposed models with the R factor defined as [4,16,18,22,36],

(29) $$R\left(\Delta \xi \right)=\frac{|J_{Q}^{R}\left(\Delta \xi \right)-J_{Q}^{L}\left(-\Delta \xi \right)|}{|J_{Q}^{R}\left(\Delta \xi \right)+J_{Q}^{L}\left(-\Delta \xi \right)|},$$

where $\Delta \xi =\xi_{R}-\xi_{L}$ is identified as the temperature gradient, for $\xi_{R}>\xi_{L}$. With Δξ>0, i.e., $\xi_{R(L)}\equiv \xi_{hot(cold)}$, the heat current flows from *R* to *L*, fulfilling $J_{Q}^{R}>0$, and if we exchange temperatures of the bath, then the temperature gradient becomes −Δξ or $\xi_{R(L)}\equiv \xi_{cold(hot)}$ and, consequently, heat flows from *L* to *R*, satisfying $J_{Q}^{L}>0$. Now, if the heat current vanishes upon reversing the temperature gradient, i.e., $|J_{Q}|$ is finite in one direction but null in the other, then complete rectification is achieved with R→1, irrespective of the value of Δξ, whereas R→0 corresponds to no rectification, i.e., no change in $|J_{Q}|$ upon inverting the temperature gradient. So, the current asymmetries in two directions are the primary criteria behind positive rectification: (i) It is clear from Figure 4 that for a given Δξ, $|J_{Q}|$ depends on |Δχ|. If $\chi_{hot}=\chi_{cold}$, or $\Delta \chi =\chi_{hot}-\chi_{cold}=0$, the heat currents will be symmetric in both ways; therefore, R=0. (ii) From Figure 6a, we find that the variation of $|J_{Q}|$ w.r.t $\chi_{\alpha }$, is completely symmetric about the magic mean −0.5, i.e., $|J_{Q}\left(\chi_{\alpha }\right)\left|=\right|J_{Q}\left(-1-\chi_{\alpha }\right)|$. As a result, following Equation (29) and Figure 6a, we find if $\chi_{hot}=-1-\chi_{cold}$ or $\bar{\chi }=\frac{1}{2}\left(\chi_{hot}+\chi_{cold}\right)=-0.5$, then $J_{Q}^{R}\left(\Delta \xi \right)=J_{Q}^{L}\left(-\Delta \xi \right)$, yielding R=0. Thus, R can be zero; either (i) the difference between the effective tunneling barriers Δχ=0, or (ii) the mean effective tunneling barrier is equal to the magic mean ($\bar{\chi }=-0.5$); otherwise, rectification occurs.

Once |Δχ|≠0 and $\bar{\chi }\neq -0.5$, effective tunneling barriers $\chi_{L(R)}$ make dissimilar effects on $|J_{Q}|$ upon reversing the temperature gradient and, consequently, R becomes nonzero. For a fixed value of |Δξ|, R increases as Δχ shifts from zero and approaches one as $\bar{\chi }$ significantly deviates from the magic mean [Figure 6b]. The reason behind this, for smaller |Δχ|, the effect of tunneling barriers on $|J_{Q}|$ are comparable upon reversing the temperature gradient, yielding partial rectification (0<R<1). However, with the increase in |Δχ|, the effect of $\chi_{L(R)}$ is no longer compatible and it creates larger heat current asymmetry between the two directions, leading to complete rectification behavior with R→1. As the heat current is symmetric about $\bar{\chi }=-0.5$, we only consider positive variations of $\bar{\chi }$ in Figure 6b. Moreover, variations of R with |Δχ| depend on the value of |Δξ|; larger Δξ implies better rectification. As $\xi_{cold}$ is less than $\xi_{hot}$ and the transition cycle is always initiated by the cold bath, the heat current executes stronger dependence on $\xi_{cold}$ than $\xi_{hot}$ for a given change in |Δξ|. Thus, the variation of R with |Δχ| yields an appreciable change when a given |Δξ| is altered due to the change in $\xi_{cold}$ than $\xi_{hot}$ [Figure 6c: Main]. So, the complete rectification is more favorable when $\xi_{cold}\ll 1$ [Figure 6a]. We may have complete rectification even without considering the absolute temperature of the cold bath close to zero, as we can vary $\xi_{cold}=k_{B}T/U$ by changing *U* and keeping *T* finite. Finally, Figure 6c (Inset) shows that heat currents become $∼10^{-6}$ times smaller upon reversing the temperature gradient, which corresponds to the R≈1 curve in Figure 6b.

## 7. Conclusions

To conclude, the present model can be implemented as an efficient thermal switch as well as a thermal rectifier to modulate the heat current close to ideal rectification, even at arbitrarily low-temperature differences between the heat reservoirs. Independent of the details of the system, the heat flow and heat rectification are characterized by a small set of universal parameters. The position of the maximum heat current at the *magic mean* “−0.5” in terms of dimensionless physical parameters, is the major finding of the domain analysis scheme presented here. The magic mean is robust and *universal* in the sense that it is invariant w.r.t the variation of any other system or bath parameter, and it truly reflects the impact of chemical potential to decide the magnitudes of the heat current. The present protocol is unique to fermionic systems and can be applied to more complicated three or multi-terminal fermionic devices. The straightforward generalization of the present scheme to the multi-terminal set-up would involve computing the magic mean associated with the multiple fermionic reservoirs. The advantage of using quantum dot systems is that they have discrete energy levels with strong on-site Coulomb interactions, and can be simultaneously tunnel-coupled to their respective reservoirs. Their discrete energy levels provide energy-selective transport and can be tuned via the application of the external gate voltages. In view of the recent experimental advances in Coulomb-coupled quantum-dot systems [49,50,51,52], our findings will have important implications in designing novel thermal devices and opening up potential applications in controlling the thermal current at the nanoscale level.

## Data Availability

Not applicable.

## Abbreviations

The following abbreviations are used in this manuscript:

QD	quantum dot
FDF	Fermi distribution function

## Appendix A. Derivation of the Lindblad Master Equation

First, we derive the master equation for our composite quantum dot system, which is coupled with fermionic reservoirs. Let us start with the total interaction Hamiltonian given by

(A1) $$H_{T}=\sum_{\alpha =L,R}H_{T}^{\alpha }=\sum_{\alpha =L,R}\sum_{k}\left(t_{\alpha k}c_{\alpha k}^{†}d_{\alpha }+t_{\alpha k}^{*}d_{\alpha }^{†}c_{\alpha k}\right).$$

It should be noted that one cannot write the fermionic interaction Hamiltonian using a tensor product representation, since the operators involved in the tensor product commute by construction while fermionic operators anti-commute. To formally derive the master equation using tensor product representation, one needs a more formal approach in terms of the Jordon–Wigner transformation. For details, we refer to References [53,54]. In the present case, starting with Equation (A1), we can derive the von Neumann equation for the total density operator of the composite system $\rho_{T}\left(t\right)$, as

(A2) $$\frac{d}{dt}\rho_{T}\left(t\right)=-\frac{i}{\hbar }\left[H_{T}\left(t\right),\rho_{T}\left(t\right)\right].$$

Integrating the above equation following Reference [54], one obtains

(A3) $$\begin{array}{c} \frac{\partial }{\partial t}\rho_{D}\left(t\right)=\frac{1}{{\left(i\hbar \right)}^{2}}\int_{0}^{t}dt^{\prime }{\mathrm{Tr}}_{L,R}\left[H_{T}\left(t\right),\left[H_{T}\left(t^{\prime }\right),\rho_{T}\left(t^{\prime }\right)\right]\right], \end{array}$$

where we denote ${\mathrm{Tr}}_{L,R}\left\{\rho_{T}\left(t\right)\right\}=\rho_{D}\left(t\right)$ as the reduced density operator for the system and also assume that ${\mathrm{Tr}}_{L,R}\left[H_{T}\left(t\right),\rho_{T}\left(0\right)\right]=0$. Here, ${\mathrm{Tr}}_{L,R}$ refers to the trace over each bath’s degree of freedom. The reduced dynamics of the system in the weak sequential tunneling limit can then be written as [21,34,35,55]

(A4) $$\begin{array}{c} {\dot{\rho }}_{D}\left(t\right)=\frac{1}{{\left(i\hbar \right)}^{2}}\int_{0}^{\infty }dt^{\prime }{\mathrm{Tr}}_{L,R}\left[H_{T}\left(t\right),\left[H_{T}\left(t-t^{\prime }\right),\rho_{D}\left(t\right)⊗\rho_{L}⊗\rho_{R}\right]\right], \end{array}$$

where we substitute $\rho_{T}\left(t\right)=\rho_{D}\left(t\right)⊗\rho_{L}⊗\rho_{R}$. Since the bath operator obeys ${\mathrm{Tr}}_{\alpha }\left\{c_{\alpha }\left(t\right)\rho_{\alpha }\right\}=0={\mathrm{Tr}}_{\alpha }\left\{c_{\alpha }^{†}\left(t\right)\rho_{\alpha }\right\}$ for α=L,R, we obtain [27,34]

(A5) $${\mathrm{Tr}}_{L,R}\left\{\left[H_{T}^{\alpha }\left(t\right),\left[H_{T}^{\beta }\left(t-t^{\prime }\right),\rho_{D}\left(t\right)⊗\rho_{L}⊗\rho_{R}\right]\right]\right\}=0\;\;\alpha \neq \beta ;\alpha ,\beta =L,R.$$

As a result, Equation (A4) simplifies to

(A6) $${\dot{\rho }}_{D}\left(t\right)=\frac{1}{{\left(i\hbar \right)}^{2}}\sum_{\alpha =L,R}\left\{\int_{0}^{\infty }dt^{\prime }\;{\mathrm{Tr}}_{L,R}\left[H_{T}^{\alpha }\left(t\right),\left[H_{T}^{\alpha }\left(t-t^{\prime }\right),\rho_{D}\left(t\right)⊗\rho_{L}⊗\rho_{R}\right]\right]\right\}.$$

Following the standard procedure of [33,54], one can then derive the master equation

(A7) $${\dot{\rho }}_{D}\left(t\right)=L_{L}\left[\rho_{D}\left(t\right)\right]+L_{R}\left[\rho_{D}\left(t\right)\right],$$

where the Lindblad operators $L_{\alpha }\left[\rho \right]$ are given by

(A8) $$\begin{array}{ccc} L_{\alpha }\left[\rho_{D}\left(t\right)\right] & \equiv & L_{\alpha }\left[\rho \right]=\sum_{\omega_{\alpha }>0}G_{\alpha }\left(\omega_{\alpha }\right)\left[d_{\alpha }^{†}\left(\omega_{\alpha }\right)\rho d_{\alpha }\left(\omega_{\alpha }\right)-\frac{1}{2}\{\rho ,d_{\alpha }\left(\omega_{\alpha }\right)d_{\alpha }^{†}\left(\omega_{\alpha }\right)\}\right]\\ & + & G_{\alpha }\left(-\omega_{\alpha }\right)\left[d_{\alpha }\left(\omega_{\alpha }\right)\;\rho \;d_{\alpha }^{†}\left(\omega_{\alpha }\right)-\frac{1}{2}\{\rho ,d_{\alpha }^{†}\left(\omega_{\alpha }\right)d_{\alpha }\left(\omega_{\alpha }\right)\}\right]. \end{array}$$

The operator $d_{\alpha }\left(\omega_{\alpha }\right)$ assumes the form of |i〉〈j| (i≠j;i,j=1,2,3,4) and causes the transition driven by the left (right) reservoir with positive energy $\omega_{\alpha }$, such that $\omega_{L}=\omega_{21},\;\omega_{43}$ and $\omega_{R}=\omega_{31},\;\omega_{42}$. In Equation (A8), the temperature-dependent bath autocorrelation functions are given by [33,53,54],

(A9) $$\begin{array}{ccc} G_{\alpha }\left(\omega_{\alpha }\right)= & \frac{\gamma_{\alpha }}{2}f_{\alpha }\left(\omega_{\alpha }\right);\;G_{\alpha }\left(-\omega_{\alpha }\right)= & \frac{\gamma_{\alpha }}{2}\left(1-f_{\alpha }\left(\omega_{\alpha }\right)\right). \end{array}$$

So, using the above relations in Equation (A8), we obtain the final expression of the Lindbladian operator as

(A10) $$\begin{array}{ccc} L_{\alpha }\left[\rho \right] & = & \sum_{\omega_{\alpha }>0}\frac{\gamma_{\alpha }}{2}f_{\alpha }\left(\omega_{\alpha }\right)\left[d_{\alpha }^{†}\left(\omega_{\alpha }\right)\rho d_{\alpha }\left(\omega_{\alpha }\right)-\frac{1}{2}\{\rho ,d_{\alpha }\left(\omega_{\alpha }\right)d_{\alpha }^{†}\left(\omega_{\alpha }\right)\}\right]\\ & + & \frac{\gamma_{\alpha }}{2}\left(1-f_{\alpha }\left(\omega_{\alpha }\right)\right)\left[d_{\alpha }\left(\omega_{\alpha }\right)\;\rho \;d_{\alpha }^{†}\left(\omega_{\alpha }\right)-\frac{1}{2}\{\rho ,d_{\alpha }^{†}\left(\omega_{\alpha }\right)d_{\alpha }\left(\omega_{\alpha }\right)\}\right]. \end{array}$$
